# Hannes Hagström 1981–2026

**DOI:** 10.1002/ueg2.70246

**Published:** 2026-06-24

**Authors:** Jonas F. Ludvigsson

**Affiliations:** ^1^ Örebro University Hospital Örebro Sweden; ^2^ Clinical Epidemiology Karolinska Institutet Solna Sweden; ^3^ Scientific Secretary of the Swedish Society of Medicine Stockholm Sweden


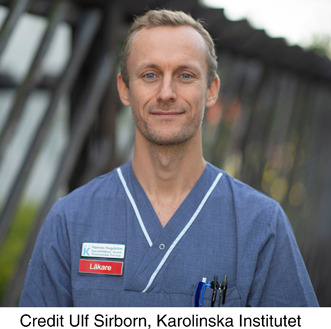


*Hannes Hagström, hepatologist and professor at Karolinska Institutet has died at the age of 45 years. He will be missed by colleagues and patients, but even more so by family and friends*.

This May it is 12 years to the day, since I received my first email from Hannes, or as he was known among fellow hepatologists: ‘Dr Hagstrom’. At the time, he was a resident and doctoral student in the team of Rolf Hultcrantz and Knut Stokkeland. The manuscript he had started writing at that time was about ‘fatty liver’ and pregnancy. In total, he would write almost 300 articles.

Hannes Hagström's career was going at record speed. He defended his thesis in 2016 and then did a postdoc at the clinical research unit ‘KEP’ at Karolinska Institutet before he established his own research group at south Karolinska University Hospital Huddinge. In 2024 he was awarded *Rising Star* at the UEGW.

Hannes had an extraordinary drive—in fact, I think I've only seen a similar drive in a handful of people during my career. Hannes created several large ‘patient cohorts’ where he studied NAFLD, later renamed MASLD.

Hannes was extremely international in his collaborations and was part of several groups that aimed to define, and increase the understanding of MASLD. He participated in guideline development for EASL, the European Association for the Study of the Liver.

A lot of Hannes' research centred around liver fibrosis development. Hannes demonstrated how fibrosis stage rather than NASH predicts mortality in MASLD [1]. His papers with Ekstedt et al. [2] and Dulai et al. [3] have each been cited more than 1000 times. Together with my team and Dr Simon, he helped explore the prognosis of biopsy‐verified MASLD [4, 5, 6]. But his achievements were not limited to observational research, he took the lead in consensus reports [7] and clinical interventions [8]. Several important findings were published in the *UEG Journal* [9]. In short, Hannes was everywhere in the liver world.

While my email communication with Hannes was about work and the 45 papers we co‐authored, our text messages also touched on other things. In recent years, Hannes became involved in the *Journal of Internal Medicine* [he was an associate editor] with its basis at Karolinska Institutet. When he asked me to peer review a manuscript for that journal earlier this year and I told him that I had to postpone the peer review because I was going to the mountains to ski with my wife, he sent me a cheerful text message and wished me great skiing. Hannes himself was a die‐hard skier.

And then in March, a few weeks later, a text message suddenly appeared on my phone asking if I wanted to join his research group for dinner in the Stockholm suburb Södermalm.

That is the Hannes Hagström I will remember.

## Conflicts of Interest

The author declares no conflicts of interest.

## Data Availability

Data sharing not applicable to this article as no datasets were generated or analysed during the current study.
